# The Impact of the Organization of Public Health Systems on the Ability of Countries to Resist the COVID-19 Pandemic: The Experience of Developed Countries of the World and Ukraine

**DOI:** 10.3390/ijerph20126106

**Published:** 2023-06-12

**Authors:** Aleksandra Kuzior, Tetiana Vasylieva, Olga Liuta, Olha Deineka, Mariia Kashcha

**Affiliations:** 1 Department of Applied Social Sciences, Faculty of Organization and Management, Silesian University of Technology, Roosevelt’s Str. 26-28, 41-800 Zabrze, Poland; 2 Department of Financial Technologies and Entrepreneurship, Sumy State University, Petropavlivska Str. 57, 40000 Sumy, Ukraine; 3 Economic Cybernetics Department, Sumy State University, Petropavlivska Str. 57, 40000 Sumy, Ukraine

**Keywords:** public health, state funding, models of healthcare financing, the integrated index of the development of medicine, the integrated index of the country’s vulnerability to COVID-19

## Abstract

The purpose of the study is to analyze the presence of functional interrelationships between the level of funding of the healthcare sector and the country’s ability to withstand any pandemic, using the example of the COVID-19 pandemic. Official indicators presented by the WHO, analytical reports by Numbeo (the world’s largest cost-of-living database), and the Global Health Security Index were used for the study. Using these indicators, the authors analyzed the following: the level of the spread of coronavirus infections in the world’s countries, the share of public expenditures on the development of the medical sphere in the GDP of the countries, and the development of the healthcare sector in 12 developed countries and Ukraine. These countries were grouped into three groups, based on the model of the organization of the healthcare sector (Beveridge model, Bismark model, Market (private) model). The Farrar–Glauber method was used to check for multicollinearity in the input dataset, and thirteen relevant indicators were selected. These indicators took part in the formation of the generalized characteristics of the country’s medical sphere and the ability to resist the pandemic. The state of readiness of countries to resist the spread of coronavirus infections was assessed using the country’s index of vulnerability to COVID-19 and the integral index of the development of medicine. Additive convolution was used in combination with sigma-limited parameterization to form an integral index of the country’s vulnerability to COVID-19 and to determine the weights of each indicator. The convolution of indicators according to the Kolmogorov–Gabor polynomial was used to construct an integral index of the development of medicine. Thus, while analyzing the ability of countries to resist the pandemic in terms of models of organization of the healthcare sector, it should be noted that none of the models demonstrated absolute effectiveness in the fight against the mass spread of COVID-19. The calculations made it possible to determine the nature of the relationship between the integral indices of the development of medicine and the vulnerability of countries to COVID-19, as well as a country’s potential ability to resist any pandemic and prevent the mass spread of infectious diseases.

## 1. Introduction

The dynamics of the epidemiological situation that has developed in the world due to the COVID-19 pandemic threatens the health of the population and the stability of the socioeconomic status in various countries. The large-scale impact of the coronavirus pandemic on all areas of society has not only delayed the achievement of Sustainable Development Goal 3, “Good health and well-being”, but also caused a prolonged interruption in the provision of essential health services in several countries, which in turn put future indicators of public health at risk [1,2]. 

An analysis of data on the spread of coronavirus infections at the beginning of 2020 proves that no country in the world was fully prepared to face the pandemic, regardless of the level of its economic development and healthcare sector development [3]. It should be noted that in some countries of the world, Italy and the USA in particular, a higher proportion of severe and extremely severe forms of the disease was registered, which caused a threat to public health, particularly in terms of the rapid depletion of human resources, as well as the reserve of beds and stocks of biomedical materials and equipment, including personal protective equipment, test systems, and medical ventilators [4,5]. 

A sharp increase in the number of patients with a coronavirus infection has become an indicator that characterizes the potential of the state to resist the COVID-19 pandemic, the degree of its readiness, and the speed of an adequate response in the conditions of an emergency in the world [6]. 

It is to be noted that the financial support of the healthcare system plays a unique role in that regard, directly affecting its sustainability.

### 1.1. Literature Review

The world’s countries’ resilience to the challenges of the COVID-19 pandemic is the focus of contemporary scholars. Identifying the influence of the specificity of the healthcare sector’s organization on countries’ ability to resist the COVID-19 pandemic has necessitated studying the results of research dedicated to this issue. The chosen research methods were structural comparative analysis, logical generalization, and scientific abstraction.

Analytical data from literary sources indicate that the healthcare sectors of most countries, including Ukraine, were not prepared to counter such a large-scale pandemic caused by COVID-19 [7]. The analysis of scientific works on the problems of organizing public health systems demonstrates a growing interest of scientists in the specifics of healthcare systems’ functioning during COVID-19 disease outbreaks. Researchers are interested in studying the planning and control of coronavirus morbidity for proper administration of healthcare. In particular, they studied the features of the healthcare sector’s functioning during the COVID-19 pandemic, healthcare policy and planning, provision of medical assistance, and ensuring equality in healthcare [8,9,10,11].

Studies focusing on the features of different components of the healthcare sectors from various countries in the context of their ability to resist the consequences of the pandemic have gained popularity. They investigated the consequences of the pandemic, which manifested differently in countries around the world, depending on the economic efficiency and resilience of the healthcare sector to COVID-19. This allowed assessing public health models’ effectiveness and efficiency in overcoming COVID-19 [1,12].

Relevant are scientific works that illuminate the interrelationship between such important characteristics as the pandemic and digitalization [13,14], the pandemic, and the transformation of labor resources in the medical field [15] in the context of the healthcare sector’s stable functioning [16,17].

Scientists have also considered the specifics of funding the healthcare sector as an important component of their effective functioning, especially in overcoming the effects of the COVID-19 pandemic [18,19].

The systematization of information sources regarding the functioning of the healthcare sector in EU countries and Ukraine indicates that the issue of financing the healthcare sector in the context of a country’s ability to resist the COVID-19 pandemic requires further research. This issue remains relevant as each country needs a healthcare sector capable of, if necessary, countering the rapid spread of future pandemics [20].

### 1.2. Analysis of the Organization of the Healthcare Sector: The Experience of Developed Countries of the World and Ukraine

While studying the problems of financial support for the development of the healthcare sector, three main models of healthcare financing are mainly distinguished:

– Budget (state) financing, which is known as the Beveridge model (Great Britain, Denmark, Norway, Finland, Sweden, Australia);
– Social compulsory health insurance, which is also called the Bismarck model (Germany, France, Switzerland);
– Private health insurance, which is known in scientific literature as a market or private model (USA) [21].

Ukraine has a budget financing system that makes it possible to attract funds from charitable foundations and receive humanitarian aid [22,23]. 

It should be noted that the specified models differ in the following characteristics: sources of funding for the development of medicine, the level of availability of medical services, the level of development of the healthcare sector, the organization of healthcare, the level of state financial support, and the influence of the state on the development of the healthcare sector. 

Table 1 summarizes the main advantages and disadvantages inherent in the researched models of financing the development of the healthcare industry.

To perform a comparative assessment of the efficiency of the functioning of healthcare systems in different countries, the World Health Organization (WHO) calculates indicators that characterize the state of a country’s healthcare sector. 

One key indicator that describes the healthcare system’s funding level is the share of public expenditures on the healthcare sector in the country’s GDP. Data on the dynamics of the level of state financing of the development of medicine in the countries’ GDP worldwide in terms of models of the organization of the healthcare system are shown in Table 2.

As shown in Table 2, the data on the share of public expenditures on the development of the healthcare sector in the GDP of the countries worldwide show that during the researched period, there was an increase in the specific weight of the public financial resources allocated to finance healthcare in the GDP in almost all developed European countries, except Denmark, where the reduction is 0.3 points. Interestingly, in countries that use the Beveridge model (except for Ukraine), the increase in the value of this indicator is the most significant and amounts to an average of 1.0 points. As for Ukraine, the trend regarding the share of public expenditures on the development of the healthcare sector in the country’s GDP is unstable and fluctuates from 4.1% in 2010 to 3.2% in 2019. The data summarized in Table 2 indicate that the share of public expenditures on healthcare in Ukraine is significantly lower compared to developed countries worldwide and does not reach 5% of the country’s GDP, the level recommended by the World Health Organization. 

While analyzing the state financing of the healthcare sector in the countries that use the Bismarck model, it can be seen that the average increase in the share of state expenditures on the development of medicine is 0.5 points. At the same time, the state financing of the healthcare system in Switzerland grew the fastest, from 9.9% in 2010 to 11.3% in 2019. 

It should be noted that in the USA, where the market (private) model of financing the healthcare sector has been implemented, the share of public expenditures in the country’s GDP is the highest. During the research period, it amounted to more than 16%. 

According to experts from the World Health Organization, the overall increase in the life expectancy of the population on the planet is primarily due to the improvement of the healthcare system. Therefore, life expectancy indicators and the mortality rate are among the primary indicators used by the WHO to assess the effectiveness of the functioning of the healthcare sector in a country [25]. 

The rapid and large-scale spread of coronavirus infections in the world made it necessary to calculate and analyze specific indicators that make it possible to determine the state of the morbidity, the criticality of the situation in the country, and the level of threats to the life and health of the population. 

As of today, the WHO publishes the following indicators of the conditions of the COVID-19 pandemic: the number of cases of infection since the beginning of the pandemic, the number of deaths caused by coronavirus infection, the number of laboratory tests performed to detect the presence of the COVID-19 virus, the number of persons who have recovered, the number of persons who recovered per day, the number of deaths per day, the number of persons who are sick at the specified date, and the number of critical cases (Table 3).

The data of the conducted analysis indicate that the most significant numbers of cases of COVID-19 per 1 million population of the country are peculiar to such countries as the USA, Great Britain, the Netherlands, and Sweden. Among the countries that have been studied, the lowest numbers of cases per 1 million population were registered in Denmark and Australia. While analyzing the rate of new cases as of 18 October 2021, it can be seen that Great Britain, Austria, the United States, and Ukraine show the most significant increase. The highest mortality rates during the pandemic were recorded in the USA, France, Sweden, and Ukraine. 

Data on the number of people who recovered during the period of the spread of coronavirus infections per 1 million people show that the Netherlands, Sweden, the USA, and France are the leaders according to this indicator. At the same time, the smallest numbers of those who have recovered are observed in Finland and Australia. 

Therefore, each country should create the appropriate potential to resist the pandemic to prevent the mass spread of infectious diseases at any time and to the full extent to ensure the safety of life and the population’s health. 

We believe that the potential of resisting a pandemic consists in ensuring such a level of development of the medical system and state support for the implementation of innovative technologies in the field of providing medical services and diagnostics, which can be quickly used for effective response and prevention of the mass spread of infectious diseases with an extremely high level of damage to the population throughout the country. 

The efficiency of the functioning of healthcare systems in countries worldwide is the subject matter considered not only by WHO experts but also by specialists of various scientific research structures based on the analysis of the relevant indicator system.

Since 2019, the Johns Hopkins Center for Medical Research, the Nuclear Threat Initiative, and the Economist Intelligence Unit have been calculating the Global Health Security Index (GHSI), which assesses global healthcare capabilities in 195 countries. The index consists of 140 questions split into six categories (prevention, detection and reporting, rapid response, healthcare systems, compliance with international requirements, and risks); it includes 34 indicators and 85 sub-indicators. Depending on the value of the Global Health Security Index, the countries that have been studied were divided into three groups:

– 67.0–83.5—the most prepared countries;
– 40.3–66.0—more prepared countries;
– 16.2–33.0—the least prepared countries.

In addition to the ranking and division of countries, the health security research method also assesses the compliance of the current situation with specific reference criteria expressed by the optimal values of the specified indicators. 

**Figure 1:**
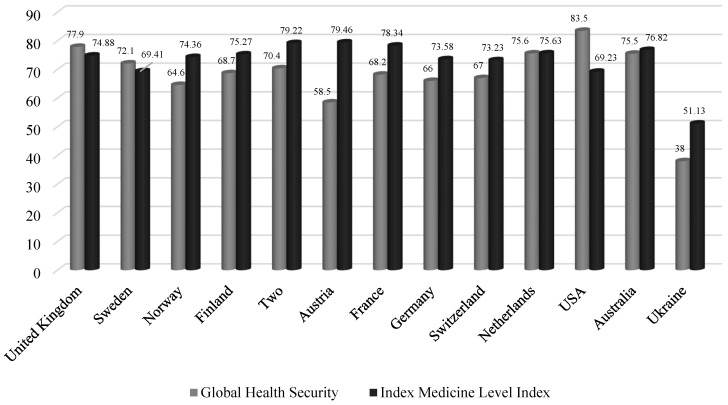
The values of the Global Health Security Index and the Medicine Level Index for countries worldwide, including Ukraine [28,29].

Interestingly, according to the results of the Global Health Security Index calculation, no country has reached the value at the level of 100% (Figure 1). This situation shows that countries worldwide were not fully prepared for the emergence and spread of epidemics and pandemics.

As part of the implementation of the Numbeo project, starting in 2018, experts have been creating a database and rating countries by level of the healthcare sector. The indicator of the level of the healthcare sector is an index of the quality of the healthcare system, which comprehensively demonstrates how developed the healthcare sector is in the country, particularly in terms of the quality of provision and availability of medical services. To create a ranking of countries by the level of the healthcare sector, experts examine the general level of quality of the healthcare system, the equipment of hospitals, the professionalism of doctors and medical staff, and the cost of medical care. Information is gained based on a survey of respondents in the corresponding countries, a comprehensive index is created based on the results of the survey. Hence, the higher the index, the higher the country’s medical care quality. 

The results of the analysis of the ranking of the countries across the world according to this indicator in 2019 indicate that the highest level of quality and availability of medical care is peculiar to Austria (79.46%), Denmark (79.22%), and France (78.34%). It should be noted that Ukraine ranks lowest among the countries that have been analyzed. 

## 2. Materials and Methods

It is worth defining complex indicators to identify the relationship between the development of the healthcare sector in the country and its ability to withstand the COVID-19 pandemic. These integral indices will be able to define the country’s vulnerability to COVID-19 and the development of the healthcare sector under different models of the healthcare system organization in the context of its ability to resist the pandemic [30]. 

Countries that are typical for the relevant models of the healthcare system organization were chosen for the study, representing the Beveridge model (Great Britain, Sweden, Norway, Finland, Denmark, Australia, and Ukraine), the Bismarck model (Austria, France, Germany, Switzerland, the Netherlands), and the market model (USA). When creating the statistical base of the study, the selected indicators were collected into the following groups [27]: 

Indicators of the prevalence of COVID-19, as of 18 October 2021 (all indicators are corrected to relative—per 1 million population of the corresponding country):

- The number of cases of infection since the beginning of the pandemic;
- The number of deaths caused by coronavirus infection;
- The number of laboratory tests performed to detect the presence of the COVID-19 virus;
- The number of persons who have recovered;
- Number of new cases of diseases per day; the number of deaths per day;
- Percentage of the vaccinated population (1 dose of vaccine) as of 18 October 2021;
- The number of persons who are sick as of 18 October 2021;
- The number of critical cases.

Basic indicators of the development of the healthcare sector as of 2019:

- The share of public expenditures on healthcare in GDP, % [25];
- Global index of health security [28];
- Ranking of countries by the level of healthcare sector [29];
- Life expectancy at birth, total (years) [25];
- Death rate, crude (per 1000 people) [25].

The Global Health Security Index is the most comprehensive, considering six categories and 37 indicators. Among the categories is the work carried out in the country on disease prevention, the speed of disease detection, the speed of response to the detected disease, the reliability of the healthcare system and the protection of medical workers, compliance with international standards, the overall value of the country’s vulnerability to epidemiological threats [28]. This indicator does not directly match the indicators we added in our study. Still, unfortunately, we do not have a way to verify the existence of hidden collinearity because we do not find all 37 indicators in public access.

For a qualitative multifactorial assessment, it is necessary to check for the absence of a close relationship between independent variables. Therefore, we checked the set of parameters that characterize the prevalence of COVID-19 for multicollinearity using the Farrar–Glauber test in the following logical sequence:

(1) Normalize the variables and find the correlation matrix of the normalized indicators;
(2) Check the presence of multicollinearity in the entire array of data using the Pearson criterion (the actual value is 52.48, which is greater than the corresponding critical value of 50.99; hence, the multicollinearity phenomenon is present in the array);
(3) Determine the multicollinearity of each variable with the array of data using Fisher’s test and Student’s t-test. The actual values obtained exceed the critical value (2.78) for such pairs of indices as the number of cases of infection since the beginning of the pandemic and the number of persons who have recovered, and the number of new cases of the disease per day.

It should be noted that there is a close relationship between the indicators of the number of people who recover and the number of infected people. Therefore, we did not consider the indicators of the number of recoveries for the entire period in further research. A similar multicollinearity testing technique was applied to the corrected array of independent variables. As a result, the hypothesis of a relationship between the data was not confirmed. 

For a set of 8 indicators of the prevalence of COVID-19, we determined an integral index (1), which provides a comprehensive assessment of the vulnerability of the country to the pandemic by using the additive convolution of the normalized indicators at the previous stage:

(1) $${COVID}_{i}=\frac{\underset{\mathrm{ik}}{m\mathrm{in}}\left(\sum_{1}^{n}x_{ik}^{*}\right)}{\sum_{1}^{n}x_{ik}^{*}}$$

where *COVID* is the integral normalized index of the vulnerability of the *i*^th^ country to the pandemic; $x_{ik}^{*}$—standardized values of pandemic prevalence indicators the *i*^th^ country, the *k*^th^ indicators.

Using the integral index, we performed a sigma-restricted parameterization, which made it possible to determine the weights of categorical variables that characterize the country’s healthcare system and calculate the impact of each factor in the overall model. 

To implement this stage, the Statistica Portable program (the Advanced Linear/Nonlinear Models module) and the GLM tool were used, in which the sigma-restricted parameterization method was chosen. The following designations were selected as a categorical indicator: the Beveridge model—1, the Bismarck model—2, and the market model—3.

The results of the sigma-restricted parametrization assessing the degree of relationship between the integrated indicator of vulnerability of countries to the pandemic (COVID) and independent indicators are contained in Table 4, together with the categorical variable of the healthcare system, which has the form shown in formula (2), and the results indicate the statistical significance of all input parameters (less than 0.05). The values according to the Fisher (F) and Student (*t*) tests, on the contrary, are large.

They are followed in descending order by the percentage of the vaccinated population (0.009), the number of patients per day (0.008), the number of new infections (0.006), the number of critical cases (0.002), the total number of diseases since the beginning of the pandemic, and the number of new deaths (0.0004). The general conclusion of model adequacy is as follows: coefficient of determination equals 0.99; multivariable correlation equals 0.99.

(2) $$\begin{array}{cc} \overset{-}{{COVID}_{i}}=0.4014 & +\;0.013TC-0.1583D-0.074T-0.112NC+0.058ND-0.192AC\\ & +\;0.193SC-0.127V0.114MBev-0.097MBism+0.104MTr \end{array}$$

where *TC* (Tot Cases) is the number of cases of infection since the beginning of the pandemic, *D* (Deaths) is the number of deaths caused by coronavirus infection, *T* (Tests) is the number of laboratory tests performed to detect the presence of the COVID-19 virus, *NC* (New Cases) is the number of new cases of diseases per day, *ND* (New Deaths) is the number of deaths per day, *AC* (Active Cases) is the number of people who are sick, *SC* (Serious, Crit) is the number of critical cases, and *V* (Vaccine) the percentage of the vaccinated population.

If the healthcare model is built according to the Beveridge model, then *MBev* = 1, *MBism* = 0, *MTr* = 0; if the healthcare model is built according to the Bismarck model, then *MBev* = 0, *MBism* = 1, *MTr* = 0; if the healthcare model is built according to the market model, then *MBev* = 0, *MBism* = 0, *MTr* = 1. 

Hence, the constructed model (2) describes 99% of the variability of the integral index from a set of parameters that characterize the degree of vulnerability of the country to the pandemic. 

For a set of indicators that are stimulators of the development of the medical field, we applied natural normalization (3) and Savage normalization for the population mortality rate (4). The applied Farrar–Glauber algorithm refutes the hypothesis of the presence of multicollinearity in the array of data from 5 indicators of the development of the healthcare system. To build an integral indicator of the development of the healthcare sector, we apply a convolution of indicators by using the Kolmogorov–Gabor polynomial (5):

(3) $$\tilde{x_{ji}}=\frac{x_{ij}-\underset{j}{min}\left\{x_{ji}\right\}}{\underset{j}{max}\left\{x_{ji}\right\}-\underset{j}{min}\left\{x_{ji}\right\}}$$

(4) $$\tilde{x_{ji}^{*}}=\frac{\underset{j}{max}\left\{x_{ji}\right\}-x_{ji}}{\underset{j}{max}\left\{x_{ji}\right\}-\underset{j}{min}\left\{x_{ji}\right\}}$$

(5) $$\begin{array}{cc} {MED}_{i}=\sum_{j=1}^{5}w_{j}\tilde{x_{ji}} & +\sum_{j_{1}=1}^{5}\sum_{j_{2}=1}^{5}w_{j}\tilde{x_{j_{1}i}}\tilde{x_{j_{2}i}}+\sum_{j_{1}=1}^{5}\sum_{j_{2}=1}^{5}\sum_{j_{3}=1}^{5}w_{j}\tilde{x_{j_{1}i}}\tilde{x_{j_{2}i}}\tilde{x_{j_{3}i}}\\ & +\sum_{j_{1}=1}^{5}\sum_{j_{2}=1}^{5}\sum_{j_{3}=1}^{5}\sum_{j_{4}=1}^{5}w_{j}\tilde{x_{j_{1}i}}\tilde{x_{j_{2}i}}\tilde{x_{j_{3}i}}\tilde{x_{j_{4}i}}+\sum_{j_{1}=1}^{5}\sum_{j_{2}=1}^{5}\sum_{j_{3}=1}^{5}\sum_{j_{4}=1}^{5}\sum_{j_{5}=1}^{5}w_{j}\tilde{x_{j_{1}i}}\tilde{x_{j_{2}i}}\tilde{x_{j_{3}i}}\tilde{x_{j_{4}i}}\tilde{x_{j_{5}i}} \end{array}$$

where $w_{j}$ is the weights of the *j*^th^ factor, and all the weights are taken as 1; $\tilde{x_{ji}}$ is the normalized value of the *j*^th^ factor of the *i*^th^ country, $j_{k}\neq j_{l}$. 

Using the integral index of the development of the healthcare sector, we conducted a sigma-limited parameterization, which made it possible to determine the weights of categorical variables characterizing the country’s healthcare system and calculate the impact of each factor in the overall model. The following designations were chosen as a categorical indicator: the Beveridge model—1, the Bismarck model—2, and the market model—3. 

The results of the sigma-restricted parametrization to assess the degree of interrelation between the integral development of medicine (MED) and independent indicators, together with the categorical variable of the healthcare system, which has the form shown in (12), indicate the statistical significance of all input parameters (less than 0.05). The values according to the Fisher (F) and Student (*t*) tests, on the contrary, are large. 

The analysis of Table 5 makes it possible to conclude that the most significant contribution to the integral index’s overall value (SS) is made by the Global Health Security Index (0.0207). It is followed in descending order by the share of public expenditure (0.0088), the ranking of countries by the level of medicine (0.0084), life expectancy (0.0056), and mortality (0.0048). The overall conclusion of model adequacy is as follows: coefficient of determination equals 0.99; multivariable correlation equals 0.99,

(6) $$\begin{array}{cc} \overset{-}{MED}=-0.787 & +\;0.5H+0.521GI+0.525LM+0.578LE+0.371DR-0.267MBev-0.269MBism\\ & -\;0.251MTr \end{array}$$

where *H* is the share of public expenditures on healthcare in GDP; *GI*—Global Health Security Index; *LM*—ranking of countries by the level of medicine; *LE*—life expectancy at birth, total (years); *DR*—death rate, crude (per 1000 people). If the healthcare model is built according to the Beveridge model, then *MBev* = 1, *MBism* = 0, *MTr* = 0; if the healthcare model is built according to the Bismarck model, then *MBev* = 0, *MBism* = 1, *MTr* = 0; if the healthcare model is built according to the market model, then *MBev* = 0, *MBism* = 0, *MTr* = 1.

Thus, the constructed model (6) describes 99% of the variability of the integral index from a set of parameters that characterize the degree of vulnerability of the country to the pandemic.

## 3. Results

The results of calculations of integral indices of the vulnerability of the country’s population to the pandemic are presented in Table 6, where a value of 0 means that the country coped best with the challenges of the pandemic and has the lowest number of deaths noted compared to the rest of the countries, whereas a value close to 1 is the opposite.

The analysis of the obtained integral indices of vulnerability to COVID-19 makes it possible to conclude that Australia was the best prepared to fight the pandemic. France, Finland, and Norway have an average level of vulnerability to coronavirus infection. According to the results of the calculations, the USA, Great Britain, Ukraine, and Austria were the most vulnerable to the spread of the pandemic. 

The normalized results of the integral index of the development of medicine are presented in Table 7.

**Figure 2:**
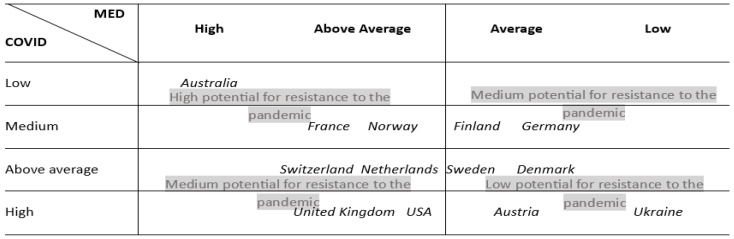
The matrix for determining the level of potential for resistance to the pandemic.

The results presented in Table 7 are spread from 0 to 1, similar to the previous table. The closer the value is to 0, the less developed the country’s health sector is compared to the other countries that participated in this research, whereas a value closer to 1 is the opposite. The analysis of Figure 2 and the obtained results of the calculation of the integral index of the development of medicine in the section of the countries worldwide makes it possible to conclude that Australia has the most developed healthcare system. The USA, Switzerland, France, the Netherlands, Great Britain, and Norway have an above-average level of development of the healthcare system. Interestingly, as evidenced by the analysis results, Ukraine demonstrates the worst level of development in the healthcare sector. 

To illustrate the relationships between the integral indices of the development of the healthcare sector and the vulnerability of countries to COVID-19, we created a matrix demonstrating the country’s potential to resist the pandemic due to the efficiency of the healthcare system (Figure 2).

Hence, if the countries are placed into the cells of the upper left corner of the matrix, this indicates that they can resist the spread of coronavirus infections and avoid significant population losses by having a sufficiently developed level of quality of the provision of medical services and the existence of appropriate financial support for the development of the healthcare sector at the beginning of the pandemic. Consequently, such countries have a reasonably high level of potential to resist the pandemic. Thus, Australia, which has a high level of the value of the integral index of the development of medicine, shows the lowest vulnerability to the spread of the pandemic. France and Norway, which have an above-average level of medical development, also demonstrate a reasonably high level of resistance to the spread of coronavirus infections. 

If, according to the results of the calculation, the countries are placed in the cells of the lower left or upper right corners, this indicates an average level of potential to resist the pandemic. Therefore, there is a need to review and improve state support and control over the development of the healthcare sector of such countries and improve the state financial policy in terms of supporting scientific and research activities in the healthcare sector. According to the calculations, Switzerland, the Netherlands, the United Kingdom, and the United States have an average potential to withstand the pandemic. 

The countries placed in the lower right corner of the matrix according to the results of the calculation of the integral indicators are characterized by a rather low level of potential to resist the pandemic. According to the results of the analysis, countries such as Sweden, Denmark, Austria, and Ukraine have insufficient capabilities to resist the spread of the virus, which requires increasing the effectiveness of state policy in the healthcare system in terms of the development of both the healthcare sector as a whole and its areas.

## 4. Conclusions

A scientometric analysis of scientific publications of the Scopus reference database in selected research areas for 2019–2023 showed that the scientific community had increased its interest in countries’ ability to withstand pandemics, including the COVID-19 pandemic. Since the problem chosen for analysis is relevant, it is advisable to study the influence of the healthcare sector’s financing level on a country’s ability to resist the spread of the pandemic. The database of the research includes 14 statistical data that describe the level of prevalence of COVID-19, the state of development of the healthcare sector, and indicators of healthcare financing in 12 European countries and Ukraine. The Farrar–Glauber Test was used to check for multicollinearity in the input dataset. For this purpose, the thirteen relevant indicators were selected. These indicators formed the generalized characteristics of the country’s healthcare sector and its ability to resist the pandemic. 

As a result of the sigma-restricted parameterization, we were able to quantitatively evaluate the influence of each indicator while calculating integral indices and single out those parameters best suited for a rapid assessment of the state of the country’s medical system and its vulnerability to a pandemic. In particular, for a quick assessment of the country’s vulnerability to COVID-19, of all the parameters, it is worth evaluating, first of all, the number of deaths per 1 million population and the number of tests performed. Similarly, for a rapid assessment of the state of the medical system, it is worth paying attention to the indicator of the Global Health Security Index and the share of public expenditures on medicine. 

The results of the construction of integral indices according to the level of development of the healthcare sector indicate that Australia has the best level (100) from the list of the studied countries. It is followed by the USA (93.2%), Switzerland (89.3%), France (88.5%), Great Britain (81.5%), and Norway (81.1%). On the contrary, Ukraine has a low value of the corresponding parameter (0%), while Germany (61.6%) and Finland (66.9%) have an average value of it. Thus, it follows that the level of development of medicine does not depend on the choice of healthcare organization model because each model has representatives among the countries that are leaders in this indicator and representatives that are outsiders. 

The results of the calculation of the integral indices regarding the vulnerability of the country to the COVID-19 pandemic showed that the leading country was once again Australia (24%), followed by France (38%), Norway (42%), and Finland (43%). The worst values were found for the USA (77%), Great Britain (91%), Austria (100%), and Ukraine (96%). Hence, we can once again conclude that the level of vulnerability to the pandemic does not depend on the choice of the healthcare organization model because each model has representatives among the countries that are leaders in this indicator and representatives that are outsiders. 

Thus, while analyzing the ability of countries to resist the pandemic in terms of models of organization of healthcare systems, it should be noted that none of the models demonstrated absolute effectiveness in the fight against the mass spread of COVID-19. At the same time, it is worth pointing out that the Beveridge model has both a model example (Australia) and a negative example (Ukraine). The Bismarck model is characterized by an average ability to resist the pandemic, confirmed by the experience of such countries as France, Norway, Finland, and Germany. Therefore, using the suggested approaches will contribute to the timely diagnosis of a country’s potential to resist the pandemic and the development and effective implementation of appropriate measures to strengthen it.

## Figures and Tables

**Table 1:** Comparative characteristics of models of healthcare financing [24].

Model	State Spending on Healthcare in GDP, %	Advantages	Disadvantages
Beveridge	6–10	the types and prices of medical services are virtually unchanged, as they are determined and controlled by the state; medical services and medical assistance are available to the entire population of the state; technologies for financing healthcare institutions and paying labor in the medical field are quite simple	lack of market instruments; stimulation of economic efficiency in the medical field; low motivation to improve the quality of medical services and medical care; the need for significant budgetary resources to finance the development of the medical field
Bismarck	10–13	high quality of medical services; redistribution of financial resources depending on the needs of the medical field; availability of medical care for all segments of the population; joint payment of medical care	significant costs for maintaining the insurance infrastructure; extensive system of administrative management; financing of healthcare institutions according to complex schemes
Market (private)	more than 10	stimulating the development of innovative technologies in the medical field; stimulating the improvement of the quality of medical services; stimulation of the intensive activity of medical workers; mobility of financial resources	due to the unregulated market of medical services, uneven access to medical care by representatives of different strata of the population; high cost of medical services; significant expenditure of the people on the development of the healthcare sector; the existence of unfair competition between doctors; lack of state control over the definition of the development of priority areas of healthcare development

**Table 2:** Dynamics of the share of public expenditures on the development of the medical sphere in the GDP of the countries of the world in terms of healthcare financing models, % [25,26].

Country	2010	2011	2012	2013	2014	2015	2016	2017	2018	2019
Beveridge model										
United Kingdom	9.8	9.8	9.9	10.0	10.0	9.9	9.9	9.8	9.9	10.2
Denmark	10.3	10.2	10.2	10.2	10.2	10.2	10.1	10.0	10.1	10.0
Norway	8.9	8.8	8.8	8.9	9.3	10.1	10.6	10.3	10.0	10.5
Finland	9.1	9.2	9.6	9.8	9.8	9.6	9.4	9.1	9.0	9.2
Sweden	8.3	10.4	10.7	10.9	10.9	10.8	10.9	10.8	10.9	10.9
Australia	8.4	8.5	8.7	8.8	9.0	9.3	9.2	9.3	9.2	9.4
Ukraine	4.1	3.7	4.1	4.2	3.6	3.6	3.2	3.4	3.3	3.2
Bismarck model										
Austria	10.2	10.0	10.2	10.3	10.4	10.4	10.4	10.4	10.3	10.4
France	11.2	11.2	11.3	11.4	11.5	11.4	11.5	11.3	11.2	11.1
Germany	11.1	10.8	10.9	11.0	11.0	11.2	11.2	11.3	11.5	11.7
Netherlands	10.2	10.2	10.5	10.6	10.6	10.3	10.3	10.1	10.0	10.2
Switzerland	9.9	10.0	10.2	10.5	10.6	11.0	11.3	11.5	11.2	11.3
Market (private) model										
USA	16.3	16.2	16.2	16.1	16.3	16.5	16.8	16.8	16.7	16.8

**Table 3:** Indicators characterizing the spread of coronavirus infections in the world’s countries as of 18 October 2021 [27].

Country	Number of Infection Cases since the Outbreak of the Pandemic	The Number of People Who Are Sick as of 18 October 2021	Number of Critical Cases	Number of Persons Who Have Recovered	Number of Persons Who Have Recovered per Day	Number of Deaths Caused by COVID-19	Number of Deaths per Day	Number of Tests Carried out to Detect the Presence of COVID-19
United Kingdom	8,272,883	1,369,174	780	6,765,629	41,288	13,808	136	316,222,267
Sweden	1,161,264	1962	29	1,126,758	2097	14,886	0	12,879,376
Norway	195,029	105,193	16	88,952	0	884	13	7,987,971
Finland	148,672	101,563	36	46	0	1109	9	7,238,282
Denmark	36,584	7448	18	355,718	537	2674	0	84,475,994
Austria	766,542	19,842	213	735,565	2128	11,135	15	91,294,829
France	7,069,089	91,344	12	6,860,572	6161	117,173	23	146,046,715
Germany	4,354,487	142,822	1336	4,116,400	101	95,265	82	73,348,901
Switzerland	852,665	38,509	132	802,995	606	11,161	5	11,261,111
Netherlands	2,033,005	6034	147	1,954,438	1733	18,227	12	17,632,552
USA	45,547,920	9,700,690	16,141	35,107,452	10,777	739,778	1819	664,075,307
Australia	133,446	26,372	296	105,596	2125	1478	17	40,307,863
Ukraine	2,578,394	21,451	177	2,304,361	6462	59,523	471	13,379,666

**Table 4:** Evaluation of the parameters of the integral index of the vulnerability of countries (COVID).

	COVID—Param.	COVID—Std. Err	SS	F	*t*	*p*
Intercept	0.40131	0.00734	0.05017	2893.69	54.879	0.0023
Tot Cases/1 M	0.01352	0.00613	0.00042	30.44	5.623	0.0412
Deaths/1 M	−0.15870	0.00954	0.01024	628.44	−24.074	0.0109
Tests/1 M	−0.07372	0.00241	0.01911	1112.33	−32.987	0.0018
New Cases/1 M	−0.11290	0.00612	0.00571	378.81	−18.657	0.0013
New Death/1 M	0.05830	0.01263	0.00042	26.70	5.450	0.0351
Active Cases/1 M	−0.19248	0.00889	0.00831	490.12	−22.093	0.0023
Serious, Crit/1 M	0.19935	0.01667	0.00231	141.42	11.657	0.0067
Vaccine/1 M	−0.12704	0.01454	0.00944	26.95	5.191	0.0041
Model 1	0.113780	0.015606			7.245	0.0128
Model 2	−0.09672	0.01391			−7.695	0.0113
Model 3	0.10361	0.01518			7.281	0.0156

**Table 5:** The evaluation of the parameters of the integral index in medicine development (Med).

	Med—Param.	Med—Std. Err	SS	F	*t*	*p*
Intercept	−0.7873	0.0483	0.0074	266.1405	−16.3138	0.0001
The share of public spending on healthcare in GDP	0.4997	0.0280	0.0088	317.7969	17.8269	0.0001
The global index of health security	0.5210	0.0191	0.0207	747.7551	27.3451	0.0000
Ranking of countries by level of medicine	0.5251	0.0301	0.0084	303.5455	17.4226	0.0001
Life expectancy at birth, total (years)	0.5777	0.0404	0.0056	204.0220	14.2836	0.0001
Death rate, crude (per 1.000 people)	0.3705	0.0280	0.0048	174.4985	13.2098	0.0002
Model 1	−0.2669	0.0147			−18.1791	0.0001
Model 2	−0.2693	0.0180			−14.9381	0.0001
Model 3	−0.2510	0.0169			−14.8119	0.0001

**Table 6:** Integral index of the country’s vulnerability to COVID-19.

Country	COVID *	Country	COVID *
United Kingdom	0.915	Germany	0.489
Sweden	0.502	Switzerland	0.584
Norway	0.421	Netherlands	0.574
Finland	0.431	USA	0.772
Denmark	0.565	Australia	0.241
Austria	1.000	Ukraine	0.956
France	0.379		

* COVID—integral indicator, values from 0 to 1, where a higher value corresponds to a better level of the development of the healthcare sector.

**Table 7:** Integral index of medicine development.

Country	MED *	Country	MED *
United Kingdom	0.815	Germany	0.616
Sweden	0.767	Switzerland	0.893
Norway	0.811	Netherlands	0.845
Finland	0.669	USA	0.932
Denmark	0.796	Australia	1.000
Austria	0.719	Ukraine	0.000
France	0.885		

* MED—integral indicator, values from 0 to 1, where a higher value corresponds to a better level of vulnerability to the pandemic.

## Data Availability

The data presented in this study are available on request from the corresponding author.

## References

[1] Lyeonov S., Bilan S., Yarovenko H., Ostasz G., Kolotilina O. (2021). Country’s health profile: Social, economic, behavioral and healthcare determinants. Econ. Sociol..

[2] Kuzior A., Mańka-Szulik M., Marszałek-Kotzur I. The impact of the Covid-19 pandemic on the economic and psychological condition of individuals and societies. Proceedings of the 37th International Business Information Management Association (IBIMA).

[3] Crowley R., Atiq O., Hilden D., Financial Profit in Medicine (2021). A Position Paper from the American College of Physicians. Ann. Intern. Med..

[4] Kuzior A., Kashcha M., Kuzmenko O., Lyeonov S., Brożek P. (2022). Public health system economic efficiency and COVID-19 resilience. Frontier DEA analysis. Int. J. Environ. Res. Public Health.

[5] Tommaso F.D. (2020). The New Italian Legislation on Corporate Governance and Business Crisis. The Impact of Covid—19 on SMEs and the Recent Rules to Mitigate the Effects. Financ. Mark. Inst. Risks.

[6] Ahmed K.M.F. (2021). Procuring Covid-19 Vaccine and the Contemporary Geopolitical Paradigm for Bangladesh. Bus. Ethics Leadersh..

[7] Kashcha M., Kwillinski A., Petrenko K. (2022). COVID-19 Vaccination Campaign: A Bibliometric Analysis. Health Econ. Manag. Rev..

[8] Lopez B.S., Alcaide A.V. (2020). Blockchain, AI and IoT to Improve Governance, Financial Management and Control of Crisis: Case Study COVID-19. SocioEcon. Chall..

[9] Kadar B., Reicher R.Z. (2020). Innovations in health care management: The effect of the pandemic on the labour market change. Mark. Manag. Innov..

[10] Kolosok S., Jakubowska A. (2020). Covid-19 and public health administration: Trends and prospects. Health Econ. Manag. Rev..

[11] Shipko A., Demikhova N., Pajak K., Motrechko V. (2021). Health management at the regional level: Multivariable performance assesment. Health Econ. Manag. Rev..

[12] Lyeonov S.V., Kuzmenko O., Koibichuk V.V., Rubanov P.M., Smiianov V.A. (2021). Behavioral, social, economic and legal dimension of the public health system of Ukrain: Descriptive, canonical and factor analysis. Wiadomosci Lekarskie.

[13] Fadel S., Rouaski K., Zakane A., Djerboua A. (2021). Estimating Climate Influence of The Potential Covid-19 Pandemic Spreading in Algeria. SocioEcon. Chall..

[14] Ober J., Karwot J. (2023). The Effect of Publicly Available COVID-19 Information on the Functioning of Society, Businesses, Government and Local Institutions: A Case Study from Poland. Int. J. Environ. Res. Public Health.

[15] Hinrichs G., Bundtzen H. (2021). Impact of COVID-19 on personal insurance sales—Evidence from Germany. Financ. Mark. Inst. Risks.

[16] International Experience of Reforming the Health Care System (Experience of the European Union Countries). http://euinfocenter.rada.gov.ua/uploads/documents/29185.pdf.

[17] World Health Organization (2021). Building Health Systems Resilience for Universal Health Coverage and Health Security during the COVID-19 Pandemic and Beyond: A Brief on the WHO Position.

[18] Shrank W., DeParle N., Gottlieb S., Powers B., Wilensky G., Orszag P. (2021). Health Costs And Financing. Challenges And Strategies for A New Administration. Health Aff..

[19] Samoilikova A., Kunev R. (2020). The impact of health care financing on the economic growth: EU countries analysis. Health Econ. Manag. Rev..

[20] Postrzednik-Lotko K. (2022). Managing quality of life in the post-pandemic period. Zesz. Nauk. Politech. Śląskiej. Organ. Zarządzanie.

[21] Klymenko M. (2017). Global experience of financing in the field of medical care. Chernihiv Scientific Journal of the Chernihiv State Institute of Economics and Management. Econ. Manag..

[22] Sabetska T. (2001). Problems and prospects of financial support of the sphere of health care of Ukraine. Econ. Soc..

[23] Ukraine: Overview of the Health Care Financing Reform 2016–2019. https://www.euro.who.int/__data/assets/pdf_file/0018/425340/WHO-WB-Joint-Report_UKR_Full-report_Web.pdf.

[24] Krynychko L., Motailo O. (2021). New approaches to financing the health care system. Asp. Public Adm..

[25] World Health Organization. https://www.who.int/data/gho/data/indicators.

[26] Budget Space for the Healthcare System in Ukraine. https://www.euro.who.int/__data/assets/pdf_file/0003/463872/UKR-Budgetary-space-for-health-ukr.pdf.

[27] The COVID-19 Coronavirus Pandemic. https://www.worldometers.info/coronavirus/.

[28] (2019). Welcome to the 2019 Global Health Security Index. https://www.ghsindex.org/.

[29] Ranking of Countries by Level of Medicine. https://www.numbeo.com/health-care/rankings_by_country.jsp.

[30] Letunovska N., Kashcha M., Dluhopolskyi O., Lyeonov S., Artyukhova N., Gąsior M., Sak-Skowron M. (2023). Health risks and country sustainability: The impact of the COVID-19 pandemic with determining cause-and-effect relationships and their transformations. Sustainability.

